# Identification and characterization of the bZIP transcription factor family and its expression in response to abiotic stresses in sesame

**DOI:** 10.1371/journal.pone.0200850

**Published:** 2018-07-16

**Authors:** Yanyan Wang, Yujuan Zhang, Rong Zhou, Komivi Dossa, Jingyin Yu, Donghua Li, Aili Liu, Marie Ali Mmadi, Xiurong Zhang, Jun You

**Affiliations:** 1 Key Laboratory of Biology and Genetic Improvement of Oil Crops, Ministry of Agriculture, Oil Crops Research Institute, Chinese Academy of Agricultural Sciences, Wuhan, China; 2 Cotton Research Center, Cotton Research Center, Shandong Academy of Agricultural Sciences, Huanghuaihai Key Laboratory of Cotton Genetic Improvement and Cultivation Physiology of the Ministry of Agriculture, Jinan, China; 3 Centre d’Etude Régional pour l’Amélioration de l’Adaptation à la Sécheresse (CERAAS), Thiès, Sénégal; National Taiwan University, TAIWAN

## Abstract

Basic leucine zipper (bZIP) gene family is one of the largest transcription factor families in plants, and members of this family play important roles in multiple biological processes such as light signaling, seed maturation, flower development as well as abiotic and biotic stress responses. Nonetheless, genome-wide comprehensive analysis of the bZIP family is lacking in the important oil crop sesame. In the present study, 63 *bZIP* genes distributed on 14 linkage groups were identified in sesame, and denominated as *SibZIP01*-*SibZIP63*. Besides, all members of SibZIP family were divided into nine groups based on the phylogenetic relationship of *Arabidopsis* bZIPs, which was further supported by the analysis of their conserved motifs and gene structures. Promoter analysis showed that all *SibZIP* genes harbor *cis*-elements related to stress responsiveness in their promoter regions. Expression analyses of *SibZIP* genes based on transcriptome data showed that these genes have different expression patterns in different tissues. Additionally, we showed that a majority of *SibZIPs* (85.71%) exhibited significant transcriptional changes in responses to abiotic stresses, including drought, waterlogging, osmotic, salt, and cold, suggesting that *SibZIPs* may play a cardinal role in the regulation of stress responses in sesame. Together, these results provide new insights into stress-responsive *SibZIP* genes and pave the way for future studies of *SibZIPs*-mediated abiotic stress response in sesame.

## Introduction

Abiotic stresses severely affect the growth and development of plants. As a sessile organism, plant has evolved complex signaling transduction pathways and various mechanisms of abiotic stress tolerance to survive in a variety of adverse conditions such as drought, salinity, waterlogging, and extreme temperature [1–3]. Transcription factors (TFs) play a vital role in abiotic stress responses signaling networks in plants through binding to promoters of specific sets of stress-responsive genes to activate or repress their expression. The basic leucine zipper (bZIP) gene family is one of the largest TF families and is characterized by highly conserved bZIP domain [4, 5]. The bZIP domain is 60 to 80 amino acids in length with two functional regions, a highly conserved basic region and a more diversified leucine zipper region [6]. The basic region is located at the N-terminus of the bZIP domain and consists of an invariant N-x7-R/K motif with approximately 16 amino acid residues, which is responsible for DNA binding and nuclear localization. The leucine zipper contains a heptad repeat of leucines or other hydrophobic amino acids that are positioned exactly nine amino acids toward the C-terminus, which is involved in specific recognition and dimerization [4–6]. In plant, bZIP transcription factors mainly recognize *cis*-acting DNA elements with an ACGT core motif, such as TACGTA (A-box), GACGTC (C-box), and CACGTG (G-box) [4, 7, 8]. However, some bZIP proteins such as GmbZIP44 from soybean recognize and bind to nonpalindromic sequences [9].

Through genome-wide analysis, members of bZIP gene family have been identified in many plant species, including 56 in humans (*Homo sapiens*) [10], 75 in *Arabidopsis* [4], 89 in rice (*Oryza sativa*) [5], 125 in maize (*Zea mays*) [11], 160 in soybean (*Glycine max*) [12], 89 in barley (*Hordeum vulgare*) [13], 92 in sorghum (*Sorghum bicolor*) [14], 69 in tomato (*Solanum lycopersicum*) [15], 55 in grapevine (*Vitis vinifera*) [16], 96 in *Brachypodium distachyon* [17], and 247 in *Brassica napus* [18]. Extensive studies through diverse functional genomics approaches in numerous plant species demonstrated that members of the bZIP TF family play crucial roles in various aspects of biological processes, including floral initiation, embryogenesis, seed maturation, and embryogenesis [4, 19, 20]. Increasing evidences have also indicated that plant bZIP proteins function as key components in response to a wide range of abiotic stresses, including drought [21, 22], high salinity [23, 24] and extreme temperature [9, 25].

The plant hormone ABA plays a central role in plants abiotic stress resistance by regulating large number of stress-responsive genes to confer abiotic stress tolerance in plants [26, 27]. The group A bZIP proteins include ABA-responsive element binding proteins (AREB) or ABRE binding factors (ABF), have been functionally characterized as important regulators in ABA-dependent gene expression and abiotic stress response [4, 27]. In *Arabidopsis*, AREB1/ABF2, AREB2/ABF4, ABF1, and ABF3 are activated by phosphorylation of subclass III SnRK2s, thereby regulating the expression of their downstream genes [28, 29]. Transgenic plants expressing *AREB2*/*ABF4* or *ABF3* increased sensitivity to ABA, altered expression of ABA/stress-regulated genes and exhibited enhanced tolerance to drought stress [21]. Rice group A bZIP proteins, such as OsbZIP23 and OsbZIP46, also play crucial roles in ABA signaling and act as positive regulators under drought stress [22, 30]. Three bZIP proteins including AtbZIP17, AtbZIP28 and AtbZIP60, which are membrane-bound endoplasmic reticulum (ER) localized bZIP proteins, play important roles in ER stress responses [31]. In addition to ER stress induced by pharmacological agents, *AtbZIP28* has also been reported to be activated by heat shock, thereby contributes to the expression of heat-responsive genes and heat tolerance [25]. In contrast to *AtbZIP28*, *AtbZIP17* is activated by salt stress, and act as a salt stress sensor/transducer in *Arabidopsis* [32]. Moreover, stress-inducible expression of the active form of *AtbZIP17*, in which the transmembrane domain and the C-terminal tail are removed, enhances the up-regulation of stress-induced genes and tolerance to salt stress [33]. Group S1 bZIPs (AtbZIP1, AtbZIP2, AtbZIP11, AtbZIP44, and AtbZIP53) preferentially form heterodimers with group C bZIPs (AtbZIP9, AtbZIP10, AtbZIP25, and AtbZIP63)[34, 35], and these bZIPs have been shown to regulate metabolic reprogramming during stress [24, 36, 37]. For example, two group S1 bZIP TFs, AtbZIP1 and AtbZIP53, that regulate carbohydrate energy metabolism and amino acid catabolism, play an important role in the root-specific response to salt stress [24]. Recently, group C/S1 bZIPs are found to regulate the expression of genes implicated in branched-chain amino acid catabolism, which is an alternative mitochondrial respiratory pathway that is crucial for plant survival during extended darkness induced carbohydrate deprivation [38].

Sesame (*Sesamum indicum* L.) is an ancient and prized oil crop, which is grown mainly in tropical and subtropical areas of the world. Sesame has high nutritional quality [39] and is widely used in baked and confectionery products. Although market demand for sesame seed continues to rise, the production of sesame is severely affected by adverse environmental stresses such as drought and waterlogging [40–42]. It is well known that *bZIP* genes play a crucial role in response to various abiotic stresses in crops. However, no genome-wide information is available for bZIP gene family in sesame. In this study, we performed genome-wide identification of bZIP gene family in sesame and comprehensively analyzed their phylogenetic relationships, conserved motifs and gene structure arrangement. Furthermore, the expression patterns of *bZIP* genes in different tissues and in response to abiotic stresses were also analyzed using publicly available transcriptome data and quantitative real-time RT-PCR (qPCR). Our results provide a perspective for further functional characterization of potentially important bZIPs that are highly involved in abiotic stress responses in sesame.

## Materials and methods

### Identification and phylogenetic analyses of the bZIP gene family in sesame

To identify all genes encoding bZIP transcription factors in sesame (*Sesamum indicum* L.), all annotated proteins were downloaded from the *Sesamum indicum* genome database (Sinbase, http://ocri-genomics.org/Sinbase/index.html)[43]. Local Hidden Markov Model (HMM) search was firstly performed based on HMM profile of the bZIP domain (PF00170) using HMMER3.0. BLAST algorithm was also used to identify the predicted sesame bZIPs with all *Arabidopsis* bZIPs as queries. Then, all resulting candidate protein sequences were further examined by SMART (http://smart.embl-heidelberg.de/) to confirm the integrity of the bZIP domain. Finally, non-redundant and confident genes were gathered and assigned as sesame bZIP genes. Multiple sequence alignments of SibZIPs and *Arabidopsis* bZIPs were performed with ClustalX (version 1.83)[44]. Subsequently, the results were used to construct unrooted phylogenetic trees based on the neighbor-joining (NJ) method in MEGA (version 5.0) program with the pairwise deletion mode [23]. The bootstrap analysis was conducted with 1000 replicates.

### Chromosomal location and duplication analysis

*SibZIP* genes were located on sesame linkage group according to their position information in the Sinbase database. The duplication pattern of *SibZIP* genes was analyzed using MCScanX software (http://chibba.pgml.uga.edu/mcscan2/) according to the previous description [45], and genes were classified into various types of duplications including segmental, tandem, proximal and dispersed under a default criterion.

### Analyses of gene structure, promoters, conserved motifs, and construction of the interaction network

The exon-intron compositions of the *SibZIP* genes were analyzed using Gene Structure Display Server (GSDS) (http://gsds.cbi.pku.edu.cn/index.php) by comparing the coding sequences with their corresponding genomic sequences from Sinbase database. The upstream 1 kb genomic DNA sequences of *SibZIP* genes were submitted to the PlantCARE database (http://bioinformatics.psb.ugent.be/webtools/plantcare/html/) to identify the putative stress-related *cis*-elements. Conserved motifs of SibZIP proteins were analyzed using MEME (Multiple Em for Motif Elicitation) v4.11.4 (http://meme-suite.org/tools/meme). Interaction network of *Arabidopsis* bZIPs was constructed using STRING database (https://string-db.org/) [46] with high confidence (score>0.9). Then, the homologs of these interactive proteins in sesame were identified with reciprocal best BLASTP analysis [47].

### Expression profiling of *SibZIP* genes using available transcriptome data

Expression analysis of *SibZIP* genes in different tissues (root, stem, flower, leaf, capsule and seed) were based on the transcriptome data derived from Sesame Functional Genomics Database (SesameFG, http://www.ncgr.ac.cn/SesameFG)[48]. Expression patterns of *SibZIP* genes under drought treatment were extracted from the transcriptome data of two sesame genotypes with contrasting drought tolerance (drought-tolerant cultivar ZZM0635 and drought-sensitive cultivar ZZM4782) (SRA accession number: SRR2886790) [49]. Expression patterns of *SibZIP* genes under waterlogging stress were obtained from transcriptome data of two sesame genotypes with contrasting tolerance to waterlogging (waterlogging-tolerant cultivar Zhongzhi No. 13 and the waterlogging-sensitive cultivar ZZM0563) (BioProject accession number: PRJNA356988) [50]. Heatmaps and hierarchical cluster analyses were constructed by MeV (MultiExperiment Viewer) [51].

### Plant materials and treatments

Sesame plants of Zhongzhi No. 13 cultivar were grown hydroponically in a growth chamber (16 h light/8 h dark cycle at 28°C). Two-week old seedlings were exposed to different abiotic stresses including osmotic, salt, and cold treatments as described in our previous study [52]. The shoots of seedlings with different treatments were harvested at 0, 2, 6 and 12 h after treatment. Shoot samples were frozen immediately in liquid nitrogen, and stored at -80°C until use.

### Quantitative real-time RT-PCR

Total RNA was extracted using the EASY spin Plus kit (Aidlab, China). The first-strand cDNAs were synthesized using the HiScript II 1st Strand cDNA Synthesis kit (Vazyme, China) following the manufacturer’s instruction. Quantitative real-time RT-PCR (qPCR) was performed on Roche LightCycler 480 real-time PCR system using the ChamQ SYBR qPCR Master Mix (Vazyme Biotech, China). Additionally, the qPCR assays were performed with three replicates and *Histone H3*.*3* gene (*SIN_1004293*) was used as endogenous control [53]. The relative expression levels were analyzed according to 2^–ΔΔCT^ method [54]. The gene-specific primers are listed in S1 Table.

## Results

### Identification of bZIP transcription factors in sesame

To identify bZIP TFs family members in sesame, a Hidden Markov Model search using the HMM profile of bZIP domains (PF00170), as well as genome-wide BLAST searches using *Arabidopsis* bZIP sequences as query, were performed to screen protein sequence data from the *Sesamum indicum* genome database (Sinbase, http://ocri-genomics.org/Sinbase/index.html)[43]. After validating the integrity of the bZIP domain using the SMART database (http://smart.embl-heidelberg.de/), a total of 63 non-redundant genes were assigned to sesame *bZIP* genes, and named from *SibZIP01* to *SibZIP63* based on their physical location in the sesame genome. The 63 predicted SibZIP proteins ranging from 131 (SibZIP58) to 780 (SibZIP11) amino acid residues in length, with an average of 339 amino acids. The gene name, gene locus ID, linkage group location, protein length, and other corresponding information of all *SibZIP* genes are shown in S2 Table.

### Distribution on chromosome and duplication events of the *SibZIP* genes

**Fig 1:**
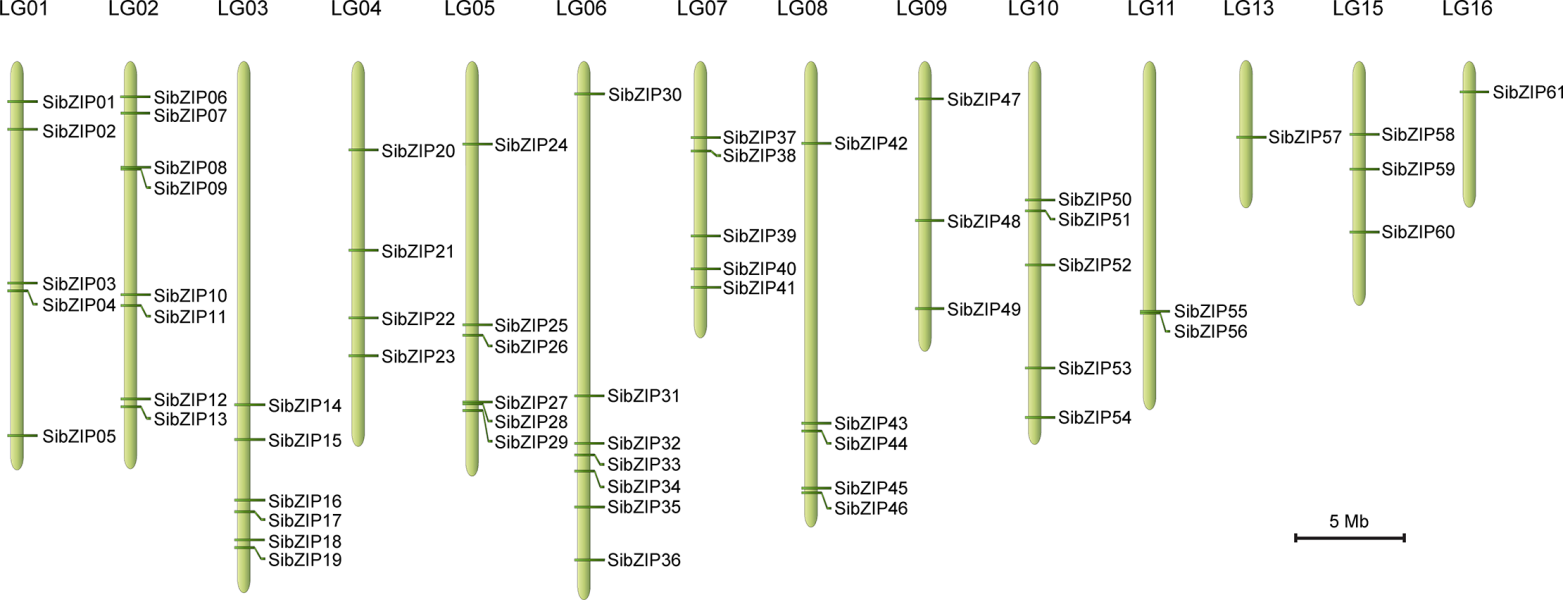
Distribution of *SibZIP* genes on sesame linkage groups. The 61 *SibZIP* genes were mapped onto 14 linkage groups (LGs) in the sesame genome. The LG number was labeled on the top of each LG. The scales were indicated the LG size of 5 Mb.

Based on the genomic position information obtained from the Sinbase database, we found that 61 *SibZIP* genes were unevenly distributed among 14 linkage groups (LGs) out of the 16 LGs of the sesame genome (Fig 1). However, other two genes (*SibZIP62* and *SibZIP63*) were distributed on the unassembled genomic scaffolds. The LG02 harbored the highest number of *SibZIP* (8 genes), followed by LG06 (7 genes). In contrast, only one *SibZIP* gene was located each on the LG13 and LG16. To understand the evolutionary mechanisms of SibZIP gene family, both tandem and segmental duplication events were further analyzed. Surprisingly, duplication in the case of bZIP gene family in sesame was confined to only segmental duplication because no tandem duplicated *SibZIP* gene pair was identified. As shown in S1 Fig, a total of 21 pairs of *bZIP* genes were located on duplicated genomic segments on 12 LGs of the sesame genome, and most of them were localized on two different linkage groups (S3 Table). These results indicate that segmental genome duplication events have contributed predominantly to the expansion of the bZIP gene family in sesame.

### Phylogenetic relationship of sesame and *Arabidopsis* bZIPs

**Fig 2:**
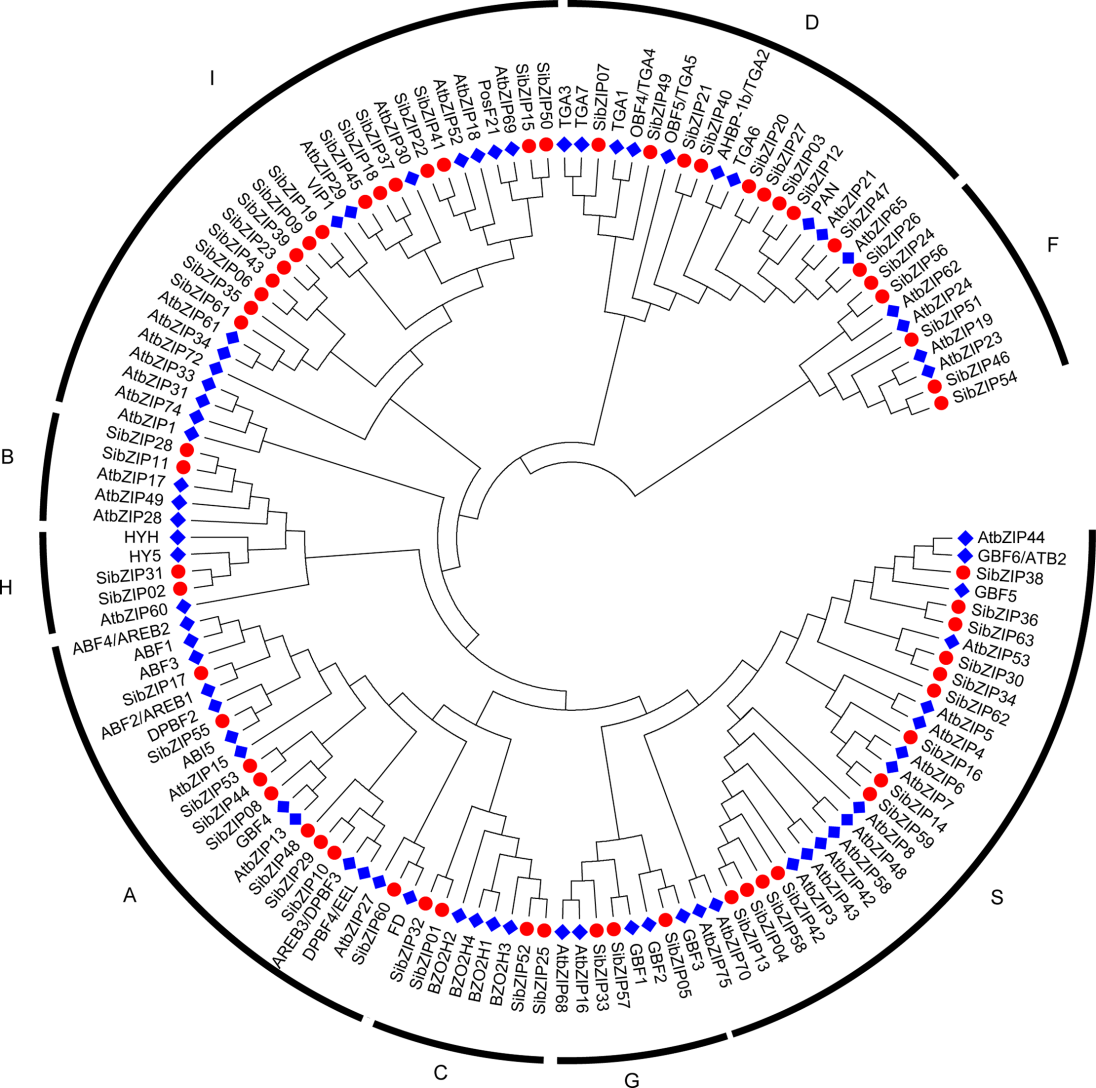
Phylogenetic tree of bZIP proteins from sesame and *Arabidopsis*. The unrooted phylogenetic tree was created based on the protein sequences of 138 bZIPs from sesame and *Arabidopsis* by MEGA5.05 using the neighbor-joining method with 1000 bootstrap replicates. The blue rhombus and red dots represent bZIPs in *Arabidopsis* and sesame, respectively.

In *Arabidopsis*, 75 AtbZIP sequences are clustered into 10 groups (A-I, and S) according to the sequence similarity. In order to understand the phylogenetic relationship of the bZIP TF family between sesame and *Arabidopsis*, an unrooted NJ tree was constructed based on protein sequences of 63 SibZIPs and 75 AtbZIPs. As shown in Fig 2, 138 bZIPs from sesame and *Arabidopsis* were divided into nine groups (A, B, C, D, F, G, H, I and S) and the groups were named following *Arabidopsis* classification. However, the group E AtbZIPs including AtbZIP34 and AtbZIP61, were clustered within the group I, which might be due to the high sequence similarity of bZIP domain between these two groups [4]. So, we integrated these two groups and designated as group I in this study. Therefore, the group I is the largest group including 15 members of SibZIP family. The group S contains 13 members. The groups D and A contain ten and nine members, respectively. In contrast, the groups B and H are the smallest clusters, each having two SibZIPs. According to the functional characterization of the bZIP subgroups in *Arabidopsis* [4], phylogeny-based functional prediction was performed for corresponding subgroups of bZIP in sesame (S4 Table).

### Gene structure and conserved motifs analyses of sesame bZIPs

**Fig 3:**
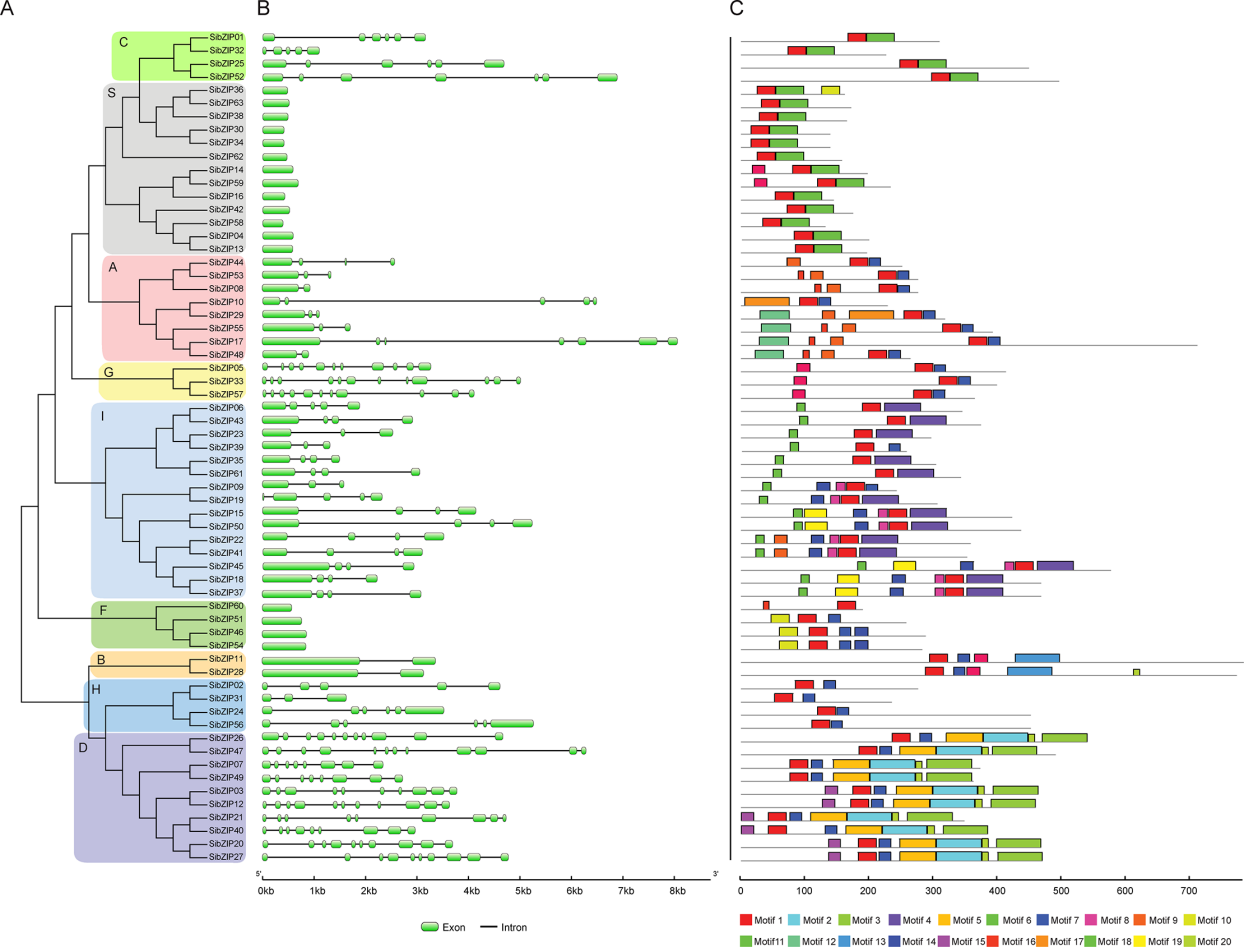
Phylogenetic relationship, gene structure, and motif composition of SibZIPs. (A) The neighbor-joining phylogenetic tree was produced by MEGA 5.05 using the neighbor-joining method with 1000 bootstrap replicates. (B) Intron/exon structures of *SibZIP* genes. The green boxes and solid lines denote exons and introns, respectively. (C) Schematic represent the conserved motifs of the SibZIPs identified by MEME. Each motif is indicated by a colored box numbered at the bottom. The details of individual motif were shown in S2 Fig.

To better understand the structural features of *SibZIP* genes, we analyzed the exon/intron organization of *SibZIPs* using Gene Structure Display Server 2.0. The result showed that the number of introns varied from 0 to 11 in *SibZIP* genes. As shown in Fig 3B, 17 *SibZIP* genes belonging to the group S and F are intronless. Groups A, B, C, H, and I have 1–6 introns, whereas, the *SibZIP* genes of the groups D and G contain the largest number of introns (7–11 introns). To obtain insight into the diversification of SibZIP proteins, conserved motifs were predicted using the MEME program. A total of 20 conserved motifs were predicted in SibZIPs, and the distribution of each motif was shown in Fig 3C. It can be observed that SibZIPs within the same group displayed similar motif compositions, which further supported the group classification. For instance, all members of the group A share motif 1, 7 and 9; group B possesses motifs 1, 7, 13 and 16; groups C and S contain motif 1 and 6; group G harbors motifs 1, 7, and 16. The details of the sequence logo of each motif were presented in S2 Fig. However, the biological function of most of these motifs is unknown. Motif 1, which is widely present in all the SibZIP TFs, was annotated as bZIP domain. In addition, motif 7 is found in six groups, and motif 6 is present in two groups. In contrast, most of the conserved motifs appeared in specific groups. For example, motif 2 and 3 were annotated as transcription factor TGA like domain, and exclusively present in group D SibZIPs along with motif 5 and 15. Besides, motif 12 is present in four members of group A, and motifs 13 only appeared in group B. These group-specific motifs may imply diverse functions of the bZIP family in sesame.

### Expression profiles of *SibZIP* genes in different tissues

**Fig 4:**
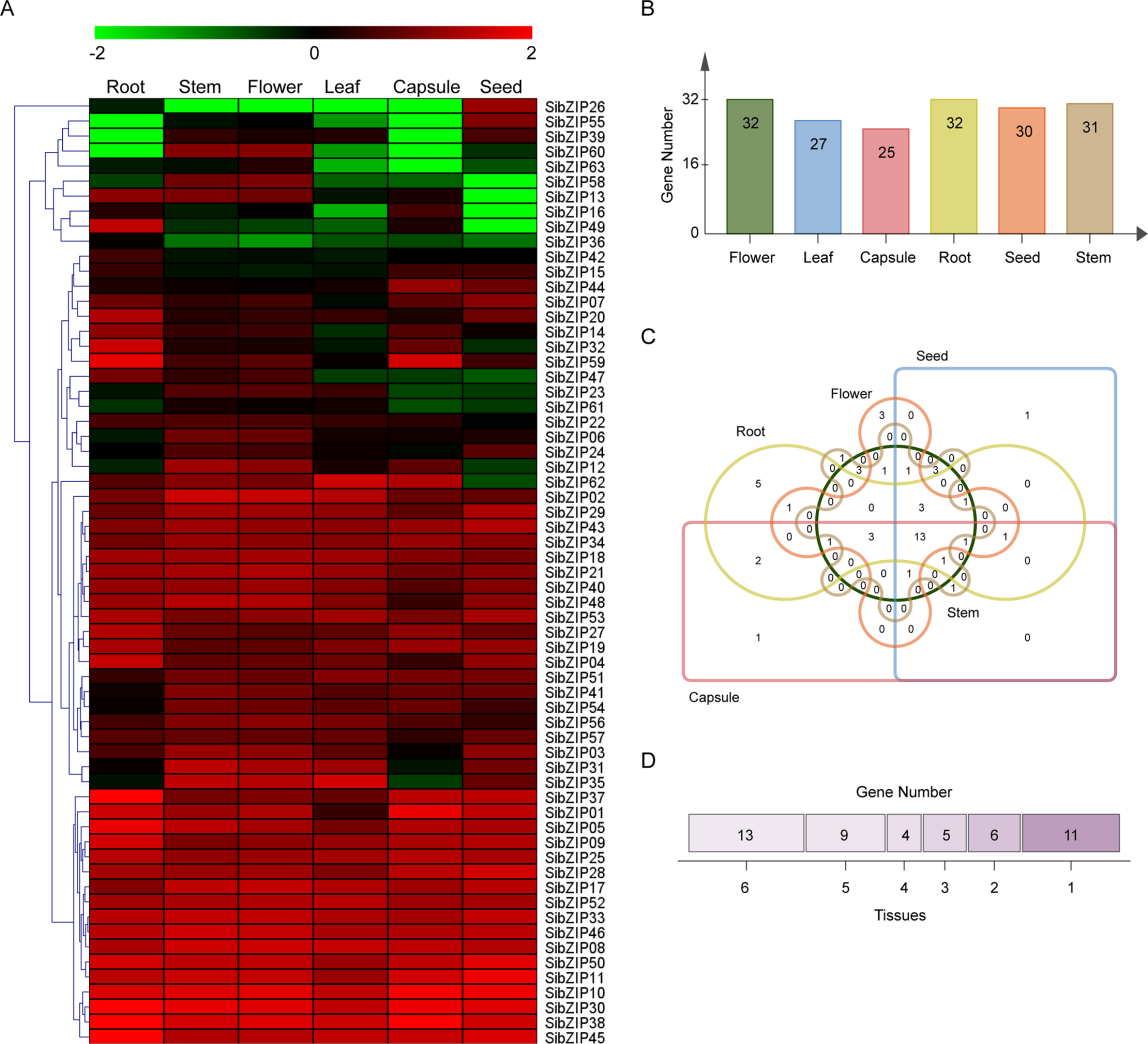
Expression profiles of the *SibZIP* genes in different tissues. (A) RNA-seq data of six tissues (root, stem, flower, leaf, capsule and seed) was used to analyze expression pattern. The heatmap was constructed using MultiExperiment Viewer with the log10-transformed RPKM values of each gene. The expression level was shown in color as the scale. (B) The number of *SibZIP* genes showed high transcriptional abundance (RPKM value > 10) in each tissue. (C) Venn diagram showing the number of overlapping *SibZIPs* that are highly expressed (RPKM value > 10) in different tissues. (D) Number of *SibZIP* genes exhibited high expression levels (RPKM value > 10): specific (1) or shared by 2, 3 … tissues.

To get insights into the transcriptional patterns and possible roles of *SibZIP* genes in sesame growth and development, transcriptome data of *SibZIPs* in six tissues/organs (root, stem, flower, leaf, capsule and seed) were obtained from Sesame Functional Genomics Database (SesameFG, http://www.ncgr.ac.cn/SesameFG) [48]. Then, heatmap generated based on the RPKM values of each *SibZIP* gene was shown in Fig 4A. All 63 *SibZIP* genes displayed very diverse expression in all tissues, in which 50.79%, 49.21%, 50.79%, 42.86%, 39.68%, and 47.62% *SibZIP* genes showed high transcriptional abundance (RPKM value > 10) in root, stem, flower, leaf, capsule and seed, respectively (Fig 4B). Further, 13 *SibZIP* genes (*SibZIP08*, *10*, *11*, *17*, *25*, *30*, *33*, *38*, *43*, *45*, *46*, *50* and *52*) were highly expressed (RPKM value > 10) in all tissues (Fig 4C and 4D). In contrast, *SibZIP15*, *16*, *22*, *23*, *36*, *39*, *42*, *61* and *63* were expressed at relatively low level (RPKM value < 5). Several *SibZIP* genes exhibited significant differences in their expression levels among different tissues. For example, *SibZIP14* and *SibZIP49* showed relatively low expression in stem, flower, leaf, capsule and seed, but high expression in root. *SibZIP26* and *SibZIP55* had high transcript abundance in seed, whereas low in other tissues. *SibZIP58* and *SibZIP60* exhibited high expression levels in stem and flower, but relatively low expression levels in root, leaf, and seed.

### Expression profiles of *SibZIP* genes under drought and waterlogging stresses

**Fig 5:**
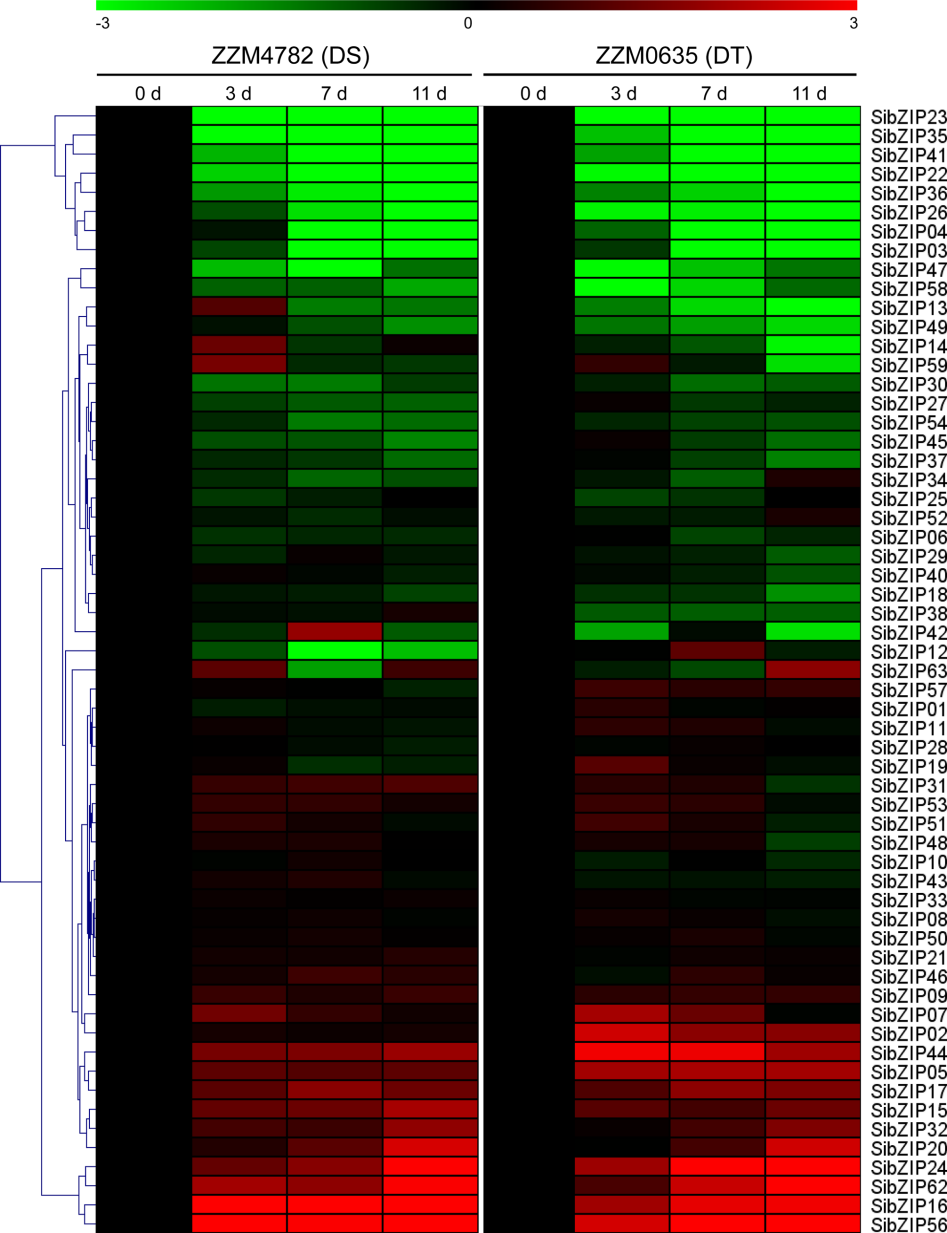
Expression profiles of *SibZIP* genes under drought stress. The heatmap was created by MultiExperiment Viewer based on the log2-transformed values of the relative expression levels of the *SibZIP* genes under drought stress in drought-sensitive cultivar (DS, ZZM4782) and drought-tolerant cultivar (DT, ZZM0635). Changes in gene expression are shown in color as the scale.

Using RNA-seq data previously developed by our group [49, 50], expression patterns of *SibZIP* genes in response to drought and waterlogging stresses were investigated in the root of sesame varieties with contrasting tolerance levels. Heatmap representing fold change of *SibZIP* genes under drought stresses showed that most of the *SibZIP* genes have similar expression pattern between drought-tolerant variety (DT, cv.ZZM0635) and drought-sensitive variety (DS, cv. ZZM4782)(Fig 5). For example, ten *SibZIPs* (*SibZIP05*, *15*, *16*, *17*, *20*, *24*, *32*, *44*, *56* and *62*) were up-regulated, whereas 12 *SibZIPs* (*SibZIP03*, *04*, *13*, *22*, *23*, *26*, *30*, *35*, *36*, *41*, *47* and *58*) were down-regulated, by drought stress in both genotypes. However, some *SibZIP* genes showed different expression pattern between DS and DT varieties and may be good candidates for drought tolerance improvement in sesame. For instance, *SibZIP02* was up-regulated in DT plants throughout the drought stress treatment, but was not significantly affected in the DS variety.

**Fig 6:**
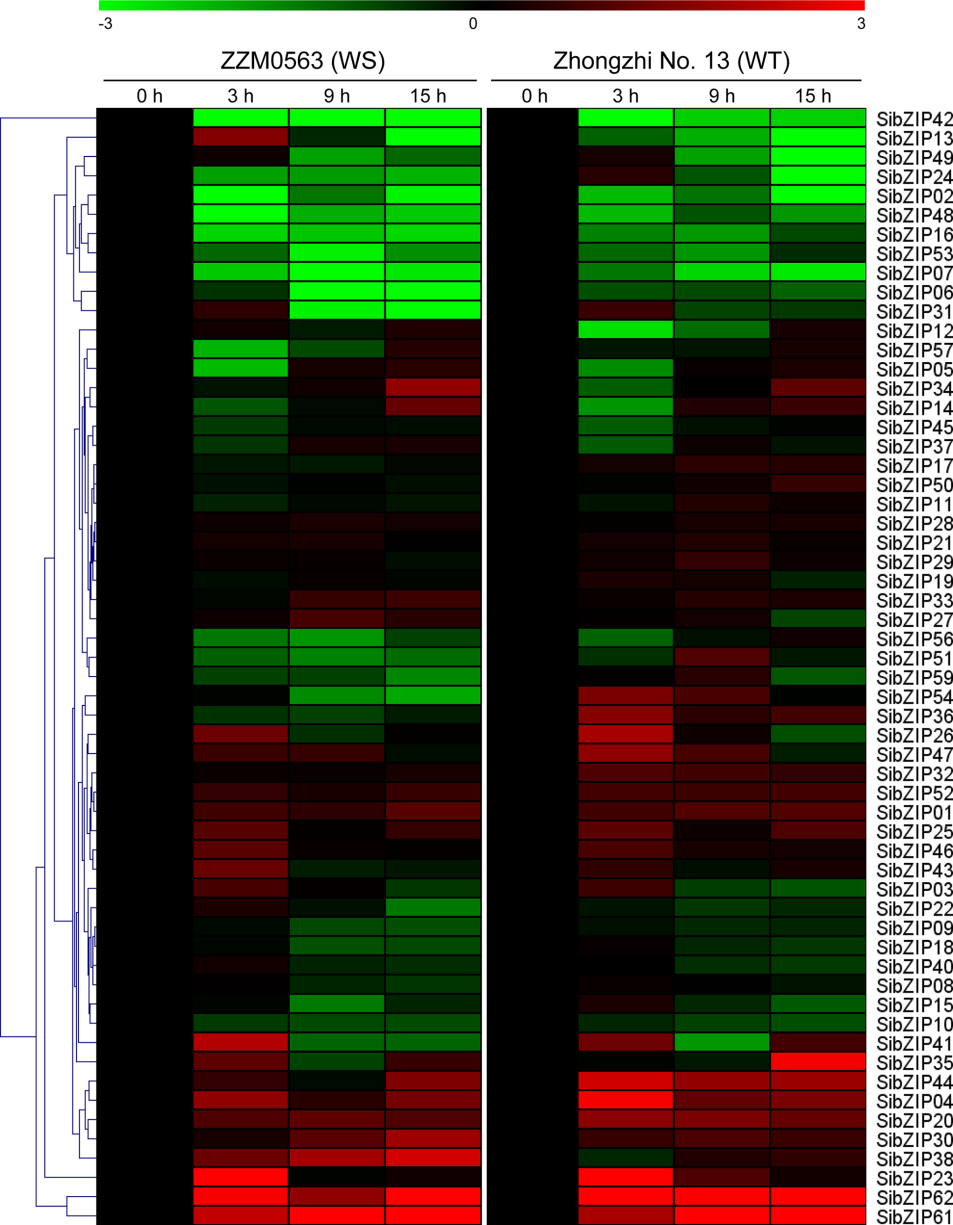
Expression profiles of *SibZIP* genes under waterlogging stress. The heatmap was created by MultiExperiment Viewer based on the log2-transformed values of the relative expression levels of the *SibZIP* genes under waterlogging stress in waterlogging-tolerant cultivar (WT, Zhongzhi No.13) and waterlogging-sensitive cultivar (WS, ZZM0563). Changes in gene expression are shown in color as the scale.

Concerning the waterlogging stress, 7.9% and 22.2% *SibZIP* genes were up-regulated and down-regulated, respectively, in waterlogging-sensitive variety (WS, cv.ZZM0563) in at least two time points (Fig 6). Similarly, 7.9% and 15.9% *SibZIP* genes were up-regulated and down-regulated, respectively, in waterlogging-tolerant variety (WT, cv. Zhongzhi No.13). Among these genes, three *SibZIPs* (*SibZIP04*, *61* and *62*) were up-regulated, whereas nine *SibZIPs* (*SibZIP02*, *06*, *07*, *16*, *24*, *42*, *48*, *49* and *53*) were down-regulated, under waterlogging stress in both genotypes. In addition, some *SibZIP* genes, such as *SibZIP36* and *SibZIP54*, showed contrasting expression patterns between WS and WT varieties. Noteworthy, *SibZIP23*, *SibZIP26* and *SibZIP41* were especially up-regulated at 3h after waterlogging stress, suggesting their possible crucial roles in early response of waterlogging stress in sesame.

### Expression profiles of *SibZIP* genes exposure to various stresses

**Fig 7:**
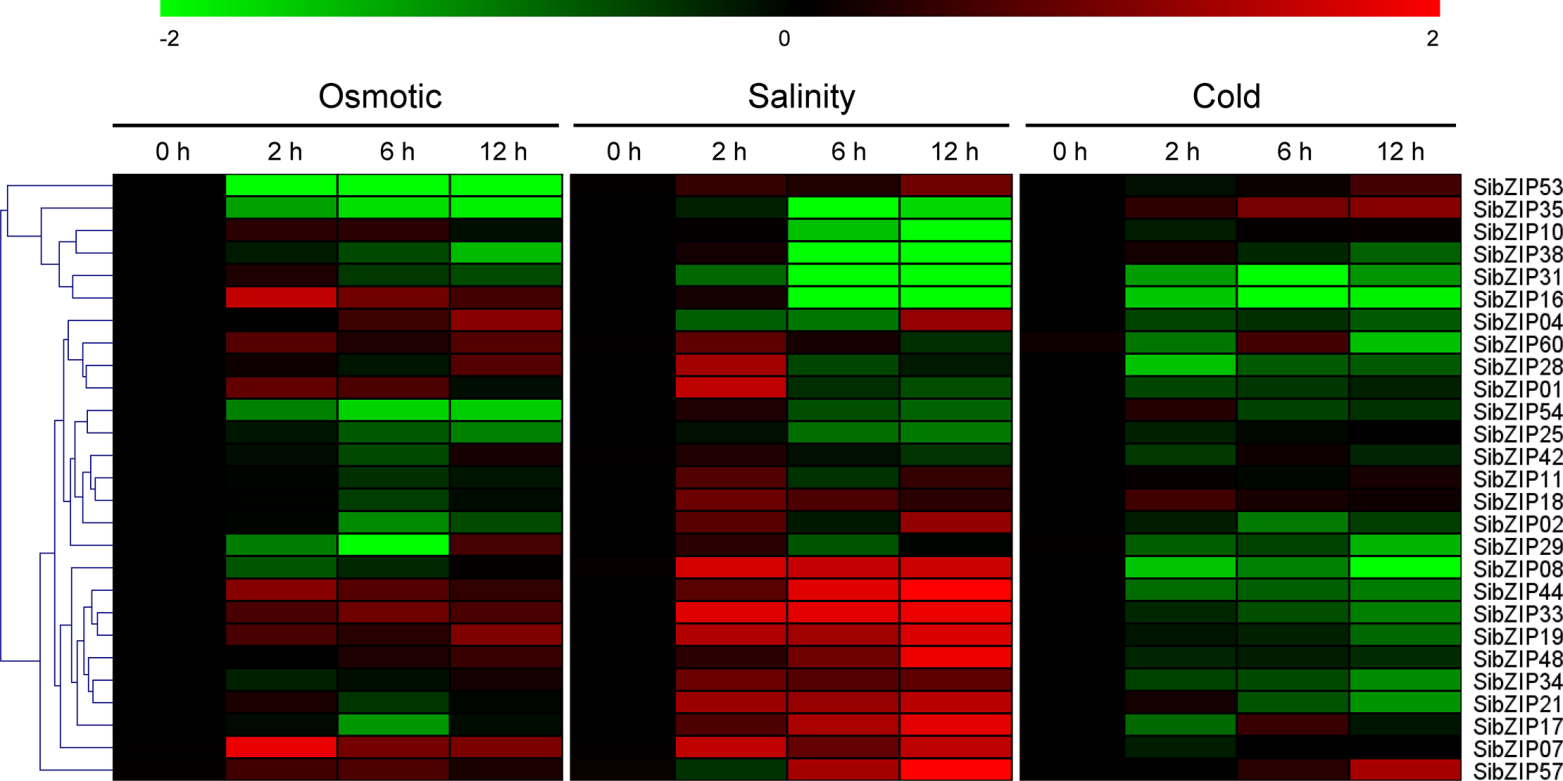
Expression profiles of *SibZIP* genes under various abiotic stresses. Two-week-old seedlings were subjected to osmotic (15% PEG 6000), salt (150 mM NaCl), and cold (4°C) stresses. The heatmap was constructed by MultiExperiment Viewer based on the log2-transformed values (mean of three replicates) of the relative expression levels of the *SibZIP* genes under various abiotic stresses. Changes in gene expression are shown in color as the scale. Original data was shown in S3 Fig.

To further uncover the possible implication of *SibZIP* genes in response to other abiotic stresses, 28 *SibZIPs* (including all members of group A, and one or two members of other groups) were selected to analyze their expression patterns under osmotic, salinity and cold stresses by qPCR. Expression of *SibZIP55*, one of the group-A *SibZIP* genes, was not detected in all samples probably due to its extremely low expression level in the tested tissue. A heatmap representation of expression changes in response to the three stresses is shown in Fig 7. Under osmotic stress, *SibZIP07*, *16* and *44* were significantly up-regulated (fold change>2, *P* < 0.01) at 2 h, while *SibZIP04* and *SibZIP19* were specifically induced at 12 h. In contrast, *SibZIP35* and *53* were down-regulated (fold change<0.5, *P* < 0.01) at all of the time points, and *SibZIP02*, *17*, *25*, *29*, *38*, *54* were repressed at one or two time points after osmotic stress treatment. Under salt treatment, eight *SibZIP* genes (including *SibZIP07*, *08*, *17*, *19*, *21*, *33*, *44*, and *57*) were significantly up-regulated (fold change > 2, *P* < 0.01) at two or three time points, whereas five *SibZIP* genes (*SibZIP10*, *16*, *31*, *35*, and *38*) were significantly down-regulated (fold change <0.5, *P* < 0.01) at 6 h and 12 h. Besides, *SibZIP01* and *SibZIP28* were specifically induced at 2 h, while *SibZIP02*, *04* and *48* were specifically induced at 12 h. Under cold treatment, almost all of the selected *SibZIP* genes tend to be down-regulated, and the expression of three *SibZIP* genes (*SibZIP08*, *16*, and *31*) was significantly repressed during the whole treatment period. However, *SibZIP35 and 57* were significantly up-regulated (fold change > 2, *P* < 0.01) at 12 h under cold stress. Taken together, we found that the expression patterns of several *SibZIP* genes are similar among different abiotic stresses (Fig 7). *SibZIP07*, *19*, *33* and *44* were up-regulated under osmotic and salt treatments. These results suggested that these genes might play a vital role in response to multiple abiotic stresses in sesame.

### Stress-related *cis*-elements in *SibZIP* promoters

To identify putative stress-responsive *cis*-elements in the promoter regions of the *SibZIP* genes, 1kb upstream promoter sequences of the *SibZIP* genes were investigated using the PlantCARE database [55]. The promoters of all *SibZIP* members contain one or more stress-related *cis*-elements, such as DRE (dehydration-responsive element), MBS (MYB binding site involved in drought-inducibility), HSE (heat shock element), LTR (low temperature-responsive element), TC-rich repeats (defense and stress-responsive element), and ABRE (ABA response element) (S5 Table). Among 63 *SibZIP* genes, 36, 34, and 34 genes have MBSs, TC-rich repeats, and HSEs, respectively. Moreover, 29 *SibZIP* genes have more than four stress-responsive *cis*-elements, and 25 *SibZIP* genes have more than three types of *cis*-elements. *SibZIP33* contains three MBS elements and may be related to drought tolerance. Similarly, *SibZIP*62 contains three HSEs, suggesting its possible function in thermal response. Furthermore, *SibZIP24*, *SibZIP33*, *SibZIP56*, and *SibZIP61* harbor various stress-related *cis*-elements, indicating these *SibZIPs* may be involved in response to multiple abiotic stresses.

### Analysis of SibZIP proteins interaction network

**Fig 8:**
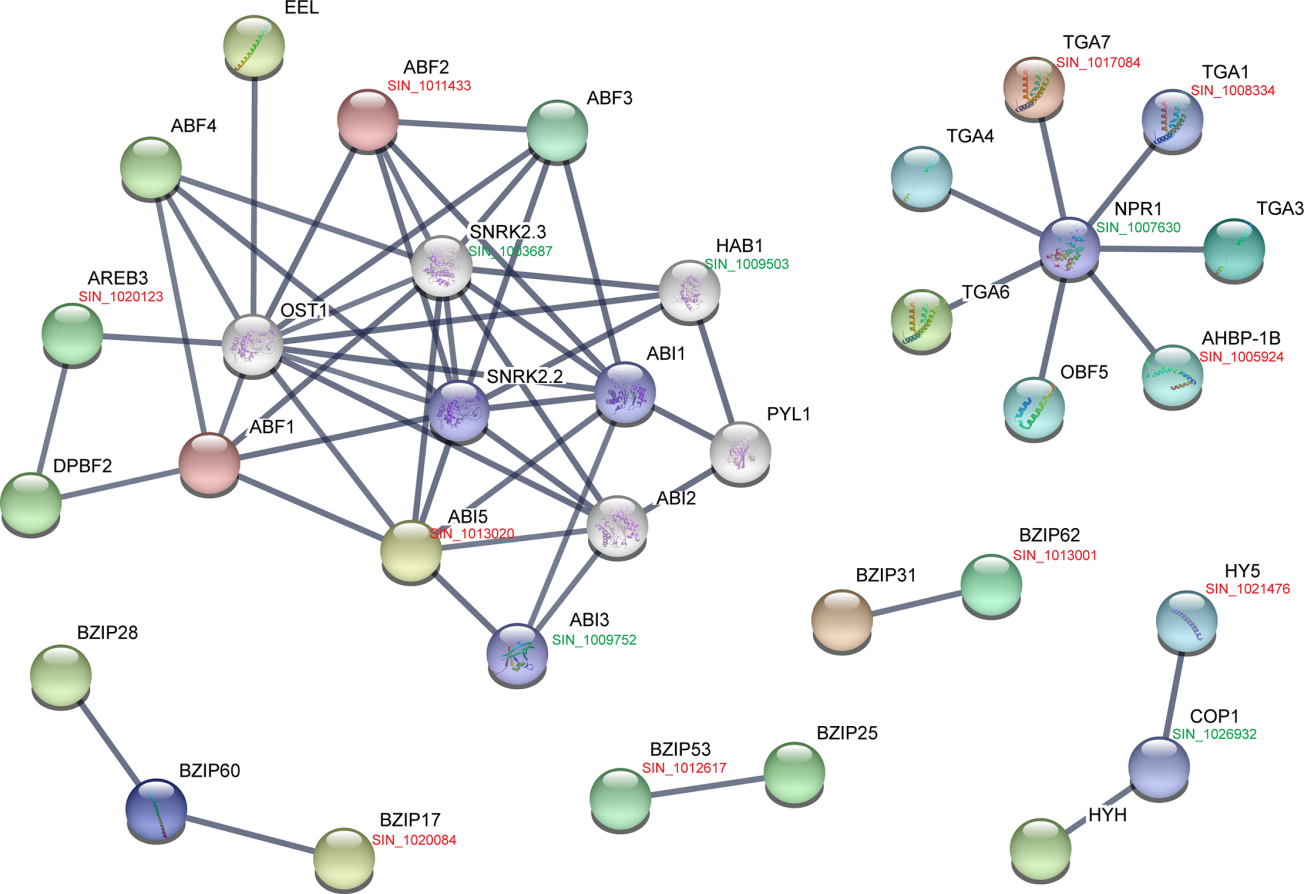
Interaction network analyses of bZIPs in *Arabidopsis* and sesame. Interaction network of *Arabidopsis* bZIPs were constructed using STRING database (https://string-db.org/) with high confidence (score > 0.9). The homologs of these interactive proteins in sesame were identified with reciprocal best BLASTP analysis. Genes marked in black show bZIPs and their interactors in *Arabidopsis*. Genes marked in red show bZIPs in sesame. Genes marked in green show the interactors of bZIPs in sesame.

The possible interaction network of sesame bZIPs based on their homology to *Arabidopsis* proteins was constructed, to identify the putative function and interaction relationship between SibZIPs and other sesame proteins. By applying STRING database, networks of *Arabidopsis* bZIPs were constructed with high confidence (score > 0.9), involving 24 bZIPs and 10 other interactive proteins, including protein phosphatase 2C, serine/threonine-protein kinase, and E3 ubiquitin ligase (Fig 8; S6 Table). Subsequently, the homologs of these proteins involved in the interaction network were identified from sesame with reciprocal best BLASTP analysis (Fig 8; S7 Table). The largest network contains eight bZIP proteins from the group A, which was associated with ABI1, ABI2, OST1, SnRK2.2, SnRK2.3 and PYL1, participating in the ABA signal pathway. Seven members of the group D interacted with NPR1, and are involved in plant defense responses. Besides, bZIP members from the groups B, C, H, and S are also involved in different interaction networks. These results offer key clues to further investigate the function of SibZIPs based on experimentally validated protein-protein interactions.

## Discussion

Sesame is an important oil crop due to its high oil, antioxidant, and protein content. Compared with other important crops, research on sesame has developed slowly, especially regarding the genetic improvement for tolerance to abiotic stresses such as waterlogging and drought [40]. Environmental stresses occurring at the vegetative and/or reproduction stage not only decrease the yield of sesame, but also affect the quality of sesame seed [42, 56]. The bZIP gene family, one of the largest transcription factor families in plants, has been reported to be involved in various biological processes, including the regulation of plant growth, development, as well as responses to abiotic and biotic stresses [4]. Recently, the genome of sesame has been sequenced [57], and this platform provides an opportunity for comprehensive analysis of sesame bZIP transcription factors from whole genome view. In total, 63 *bZIP* genes were identified in sesame. The *bZIP* genes have been identified in several plant genomes, including *Arabidopsis* [4], rice [5], maize [11], soybean [12], tomato [15], grapevine [16], *Brachypodium distachyon* [17], and *Brassica napus* [18]. Compared with these plant species, sesame contains the fewest *bZIP* genes except grapevine. Gene family expansion in plants through whole genome duplication or tandem duplications has played a major role in the evolution of functional diversity [58]. We identified 21 pairs of segmental duplicated genes, representing 66.7% of the *SibZIPs*. However, no pair of the *SibZIP* genes was identified to be arranged in tandem. Thus, we conclude that segmental duplications have played predominantly roles in the expansion of the SibZIP gene family, which is in agreement with previous studies in rice and grapevine [5, 16]. It has been reported that sesame has experienced a whole genome duplication event approximately 71 million years ago [57], which provides the opportunity for subsequent functional divergence of duplicated gene pairs. We found that some segmental duplicated *SibZIP* gene pairs, such as *SibZIP04*/*SibZIP13* and *SibZIP36*/*SibZIP38*, have different expression patterns in various organs and/or abiotic stress responses, suggesting these gene pairs have undergone functional divergence during long-term evolution.

Phylogenetic analysis show that the sesame bZIPs could be separated into 9 groups, according to the classification of bZIPs in *Arabidopsis* [4]. Except that group E bZIPs is clustered into group I in sesame duo to highly similarity of zipper motif of these two groups [4]. The phylogenetic analysis was also supported by the conserved motif and gene structure analyses. Generally, bZIPs within the same group shared similar motif architecture and tend to perform the same biological functions. For example, AtHY5 from *Arabidopsis* and OsbZIP48 from rice, which belong to the group H bZIPs, play a central role in regulating photomorphogenesis in dicot and monocot system, respectively [59]. Since none of the bZIP transcription factors has been functional characterization in sesame, we performed the phylogeny-based functional prediction of SibZIPs based on the functional characterization of corresponding bZIP subgroups in *Arabidopsis* (S4 Table). Moreover, the potential interaction networks of SibZIPs were constructed, which may provide key evidence for functional predictions of SibZIPs. In *Arabidopsis*, ABA receptors (PYR/PYL/RCAR), protein phosphatases (PP2C), protein kinases (SnRK2) and transcriptional regulators (group-A bZIP, AREB/ABFs) were confirmed as crucial components of ABA signaling [27]. Based on the interaction networks of SibZIPs, the group-A bZIPs of sesame (SibZIP17, SibZIP29 and SibZIP55), which may be triggered by SiSnRK (SIN_1003687), probably function in ABA signaling as well as regulation of stress responses and seed development. SibZIP07, SibZIP21 and SibZIP49 were clustered with *Arabidopsis* TGAs in the group D. They are predicted to interact with SiNPR1 (SIN_1007630) and may be involved in plant defense responses by activating the expression of SA-responsive genes [60]. Two group-F SibZIPs, SibZIP46 and SibZIP54, showing high similarity to AtbZIP19 and AtbZIP23 in *Arabidopsis*, were predicted to regulate the adaptation to zinc deficiency [61].

The bZIP gene family is described as involved in growth and development processes of plants, including flower development and seed maturation [4, 19]. For instance, *AtbZIP29*, which is specifically expressed in proliferative tissues, participates in leaf and root development by regulating the expression of genes involved in cell cycle and cell wall organization [62]. We analyzed the expression profiles of *SibZIP* genes in six different tissues of sesame, including root, stem, flower, leaf, capsule and seed. Although most of *SibZIP* genes were broadly expressed in all tested tissues, some *SibZIP* genes showed significant variation in their expression between different tissues, which is consistent with previous studies in rice, maize and grapevine [5, 11, 16]. Tissue-specific expressed genes including *SibZIP26* and *SibZIP55* in seed, *SibZIP14* and *SibZIP49* in root, may play key roles in specific tissue/organ development. In *Arabidopsis*, ABI5 is necessary for seed development, germination, and seedling growth [4, 63]. The gene *SibZIP55*, which is specially expressed in seed, is the homolog of the gene *ABI5* in *Arabidopsis*, suggesting its role in seed development in sesame.

Increasing evidences demonstrated that bZIP TFs have an essential function in plant abiotic stress resistance. Group-A bZIPs have been extensively examined and are reported to play significant functions in various abiotic stresses by mediating ABA signaling in *Arabidopsis* [4, 21]. A number of studies also have evaluated the function of group-A bZIP TFs in abiotic stress resistance of crop plants [22, 30]. For example, overexpression of exogenous and endogenous AREB/ABF orthologs in cotton substantially increases drought tolerance through stomatal regulation [64]. In this study, the expression level of all *SibZIP* genes under drought and waterlogging stresses were analyzed based on transcriptome data. Moreover, group-A *SibZIPs* and other *SibZIPs* from different groups were further tested under cold, osmotic and salinity stresses by qPCR. The *cis*-elements and expression pattern analysis of *SibZIP* genes indicated that *SibZIPs* are widely involved in responses to abiotic stresses, which is consistent with the results observed in other plants such as soybean, grapevine, and rice [5, 12, 16]. In total, over 80% *SibZIP* genes showed significantly transcriptional changes (> 2-fold change) after abiotic stress treatments at least one time point. Most of the group-A *bZIPs* in sesame were responsive to at least one abiotic stress condition. Notably, the expression of *SibZIP17*, homolog of *Arabidopsis ABF* gene, was up-regulated by drought and salinity stresses. In addition, *SibZIP44* containing one MBS and one ABRE, was induced by drought and waterlogging stresses. Group-D bZIPs, such as TGA1 and TGA4, not only involved in defense response to pathogens, but also identified as important regulatory factors of the nitrate response in *Arabidopsis* [65]. Moreover, overexpression of *AtTGA4* improved drought resistance and reduced nitrogen starvation in *Arabidopsis* [66]. The expression of group-D genes *SibZIP07* and *SibZIP20* were up-regulated by drought stress, consistent with the presence of MBSs and TC-rich repeats in their promoters. These results suggested their possible roles in regulating drought and low nitrogen stresses in sesame. The group-S bZIPs are transcriptionally activated after stress treatment and regulate carbon and nitrogen metabolism [4, 24, 36]. The up-regulation of four group-S *SibZIPs* (*SibZIP04*, *30*, *38* and *62*) in response to waterlogging stress suggested that they might play important roles in the metabolic reprogramming during waterlogging stress.

## Conclusions

In conclusion, we identified 63 *bZIP* genes from sesame and investigated their phylogenetic classification, conserved protein motif, and gene structure. Transcriptomic analysis revealed the constitutively or tissue-specific expressed *SibZIP* genes. Expression profiles of *SibZIP* genes under various abiotic stress treatments and protein interactional network analyses indicated that they are involved in abiotic stress signaling. Meanwhile, some important candidates for improving sesame resistance to multiple stresses were identified. Together, these data provide useful information for further functional characterization of *SibZIP* genes and extending our knowledge of SibZIPs-mediated abiotic stress response in sesame.

## Supporting information

S1 Fig Segmentally duplicated SibZIPs on sesame linkage groups. Grey lines indicated collinear blocks in whole sesame genome, and red lines indicated duplicated SibZIP gene pairs.
(TIF)

S2 Fig Sequence logos for conserved motifs identified in SibZIPs by MEME analysis. (TIF)

S3 Fig Expression profiles of *SibZIP* genes under osmotic, salt and cold. Two-week-old seedlings were subjected to osmotic (15% PEG 6000), salt (150 mM NaCl), and cold (4°C) stresses. Relative expression levels of *SibZIP* genes were analyzed by qPCR, using sesame *SiH3*.*3* gene as the internal control. Error bars indicate standard deviations (SD) based on three replicates. *P < 0.05; **P < 0.01, *t* test.
(TIF)

S1 Table List of primers used for quantitative real-time RT-PCR analysis. (XLSX)

S2 Table Characteristics of bZIP transcription factors in sesame. (XLSX)

S3 Table Segmental duplicated bZIP gene pairs within sesame genome. (XLSX)

S4 Table Phylogeny-based functional prediction of SibZIP genes based on Arabidopsis functional groups. (XLSX)

S5 Table Numbers of known stress-related *cis*-elements in the promoter regions of *SibZIP* genes. (XLSX)

S6 Table The protein interaction relationship in the bZIP-mediated interaction network in *Arabidopsis*. (XLSX)

S7 Table The homologous genes in sesame from the interaction network. (XLSX)
