# Supplementary figures and images for: Identification and characterization of the bZIP transcription factor family and its expression in response to abiotic stresses in sesame
Source: PLoS One. 2018 Jul 16;13(7):e0200850. doi: 10.1371/journal.pone.0200850 (PMC6047817; doi:10.1371/journal.pone.0200850)

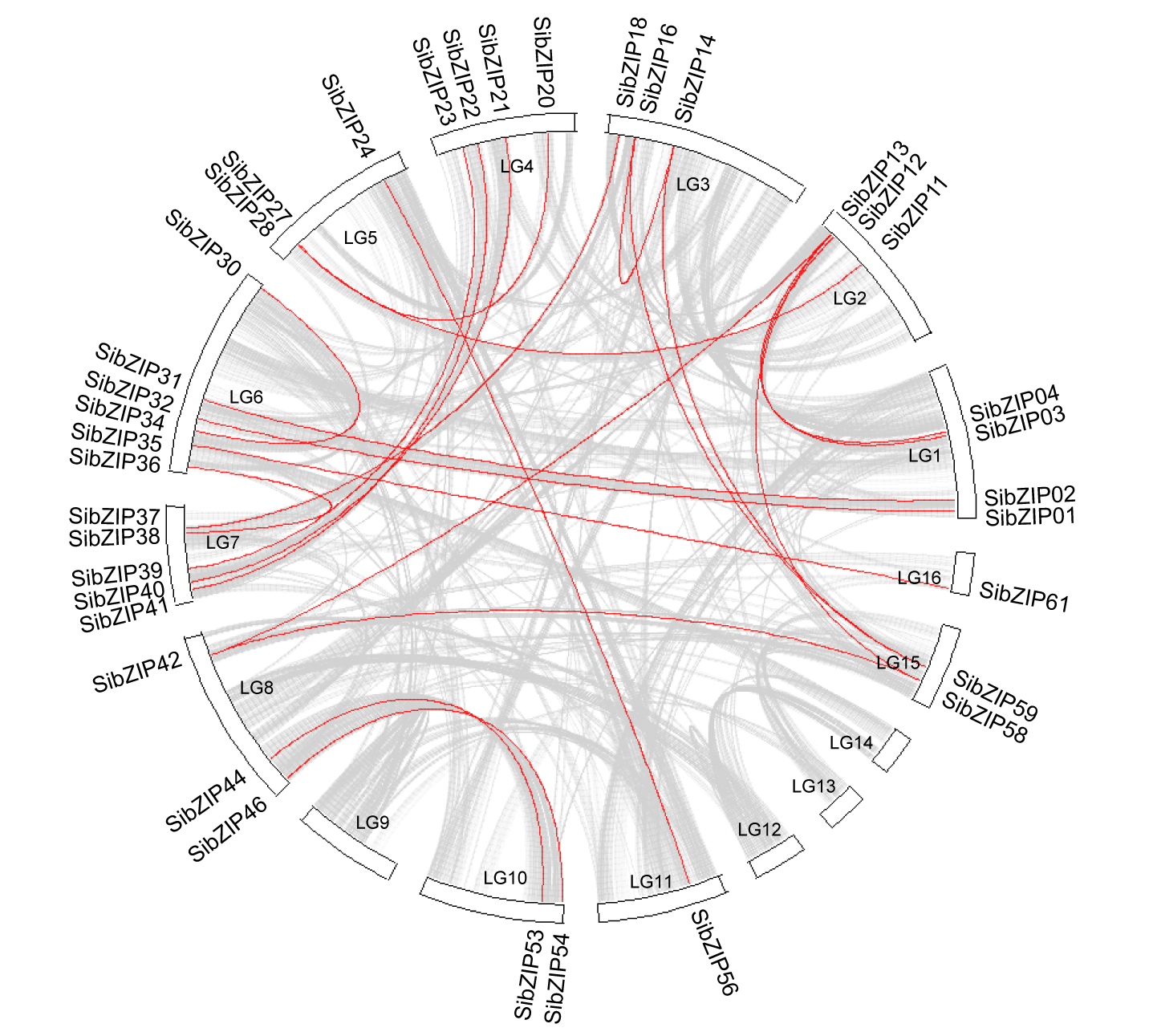

Supplement: S1 Fig — Grey lines indicated collinear blocks in whole sesame genome, and red lines indicated duplicated SibZIP gene pairs. (TIF) [file pone.0200850.s001.tif]

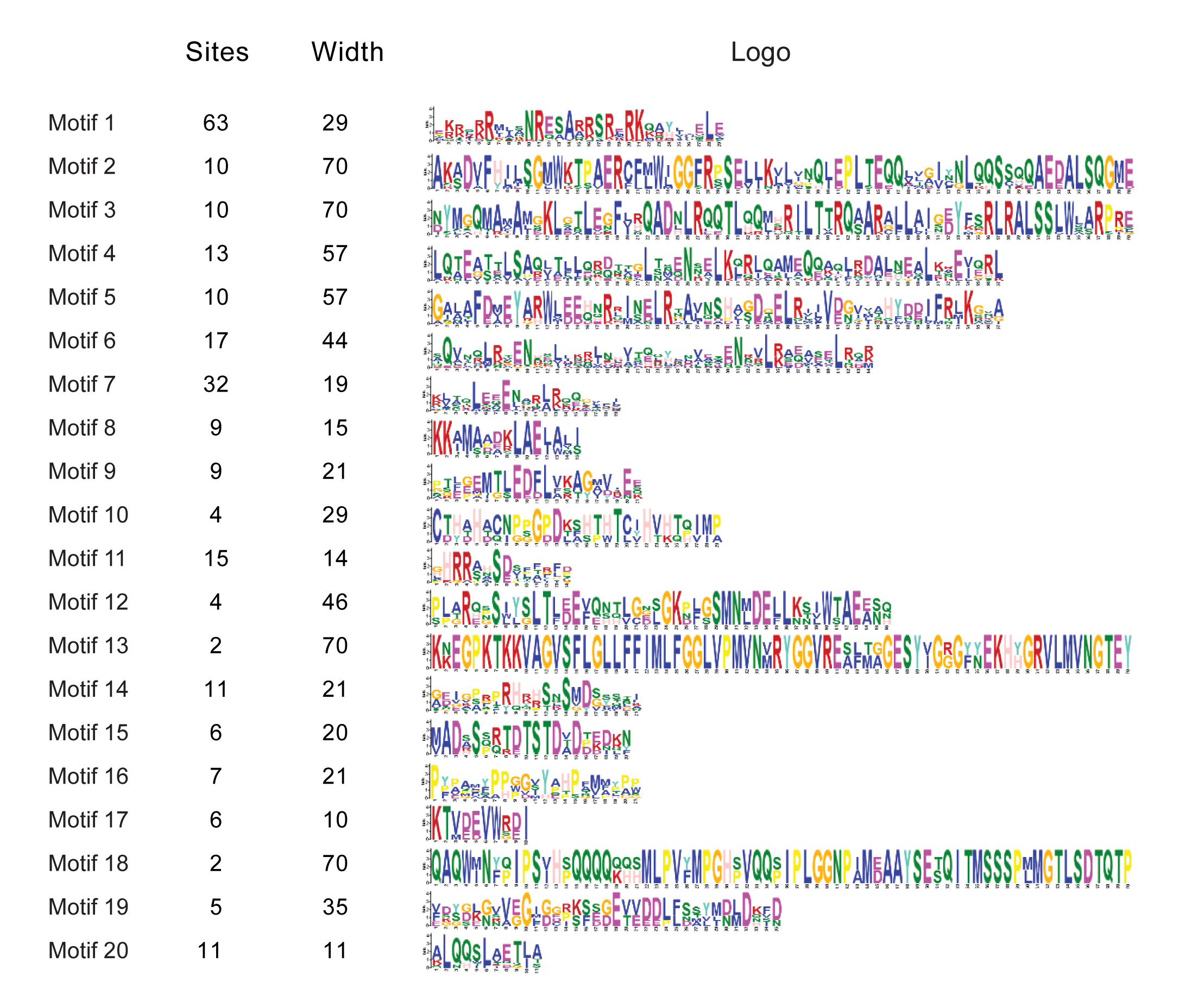

Supplement: S2 Fig — (TIF) [file pone.0200850.s002.tif]

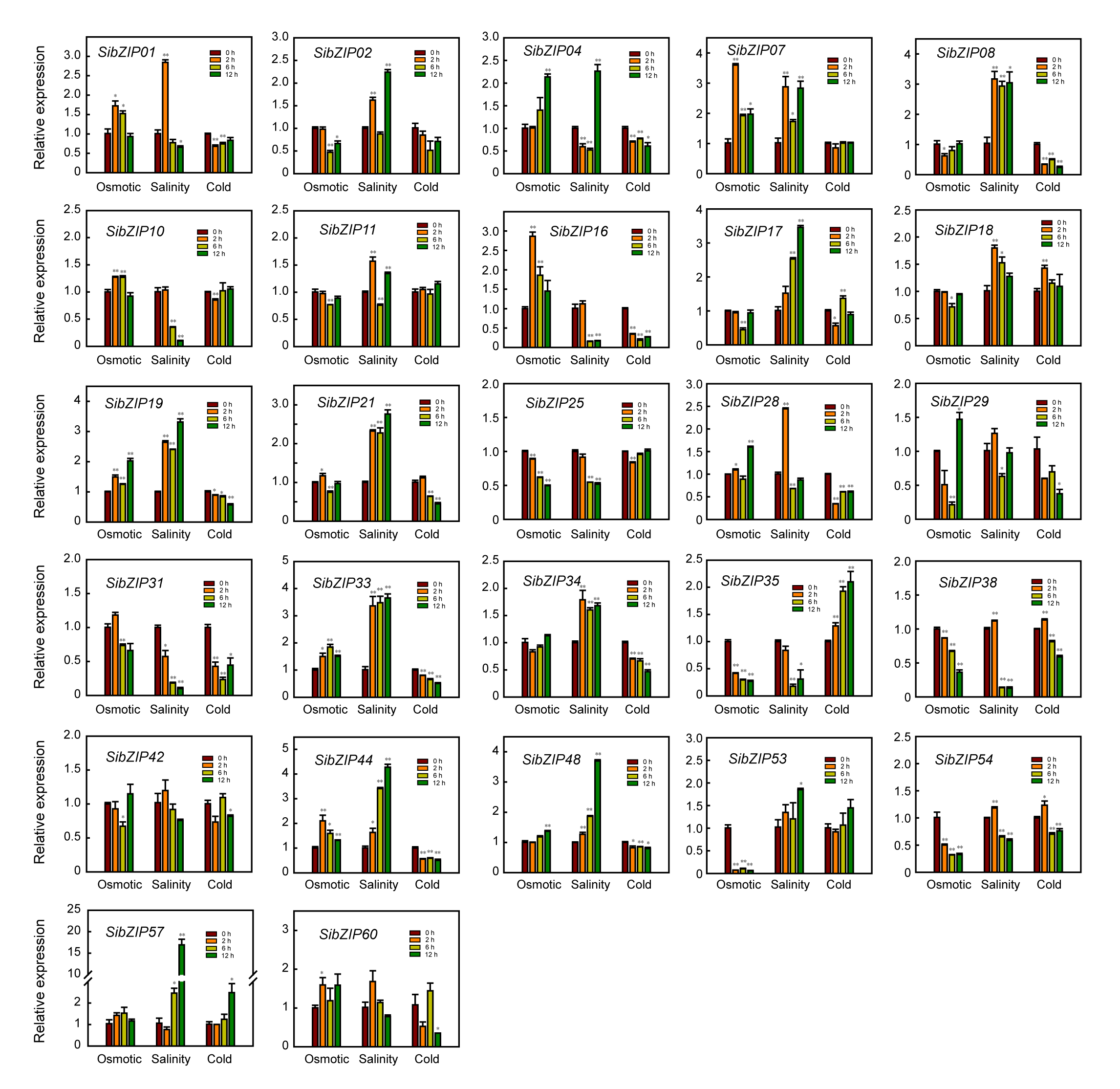

Supplement: S3 Fig — Two-week-old seedlings were subjected to osmotic (15% PEG 6000), salt (150 mM NaCl), and cold (4°C) stresses. Relative expression levels of SibZIP genes were analyzed by qPCR, using sesame SiH3.3 gene as the internal control. Error bars indicate standard deviations (SD) based on three replicates. *P < 0.05; **P < 0.01, t test. (TIF) [file pone.0200850.s003.tif]
